# Effect of Preprocedural Consumption of Banana, Plain Dark Tea, or Nothing on MRCP Image Quality in Patients With Obstructive Jaundice

**DOI:** 10.1155/bmri/6496493

**Published:** 2026-06-22

**Authors:** Abeer Kadum Abass Al-Zuhairy, Sima Radha Hamakhurshid, Sana Radha Hamakhurshid, Belan Kamal Muhammad

**Affiliations:** ^1^ Branch of Clinical Sciences, College of Medicine, University of Sulaimani, Sulaimaniyah, Iraq, univsul.edu.iq

**Keywords:** image quality, natural product, obstructive jaundice, pancreaticobiliary system

## Abstract

**Background:**

Magnetic resonance cholangiopancreatography (MRCP) is crucial in obstructive jaundice (OJ) to determine the cause, level, and extent of the obstruction.

**Objectives:**

We are aimed at comparing MRCP image qualities among patients who received different preparations.

**Methods:**

In this nonrandomized observational cross‐sectional study, 86 cases of OJ were examined at the imaging center, Sulaimani Teaching Hospital, Sulaimaniyah, Iraq, from November 2023 to April 2024. The patients were prepared before MRCP imaging by giving a solid banana, plain dark tea, or nothing (fasting for 4–6 h). Then, images were captured and compared across patients to assess their effectiveness in suppressing signals from the gastrointestinal tract (GIT) and to visualize and assess the pancreaticobiliary system.

**Results:**

Bananas had the greatest efficacy in suppressing GIT signals, followed by fasting alone and then plain dark tea (*p* ≤ 0.05). Banana consumption significantly improved main pancreatic duct (MPD) visualization (OR = 7.00, *p* = 0.021). MPD visualization improved after consuming plain dark tea compared with fasting alone. MPD was not dilated in most cases (84.9%) after all preparations were used. Age and gender are not significantly related to the appearance quality of the pancreaticobiliary system.

**Conclusions:**

Bananas are associated with GIT signal suppression, MPD visualization, and pancreaticobiliary tree dilation more than plain dark tea and fasting.

## 1. Introduction

Obstructive jaundice (OJ) represents a surgical challenge caused by cholestasis. Cholestasis is either extrahepatic or intrahepatic and causes abnormalities in liver function tests. Once these were detected, imaging techniques were encouraged to define the level of obstruction, cause, and extent [1]. Currently, available imaging modalities for OJ include transabdominal ultrasonography (US), endoscopic retrograde cholangiopancreatography (ERCP), percutaneous transhepatic cholangiography, endoscopic ultrasonography (EUS), magnetic resonance cholangiopancreatography (MRCP), and helical computed tomography (CT) [2, 3]. MRCP offers a modern imaging technique without using ionizing radiation or requiring negative oral contrast Agents 4; instead, it relies on a noninvasive, heavily T2‐weighted signal that provides detailed images of the intrahepatic and extrahepatic biliary tree, the pancreatic ductal system, and the gallbladder. It provides a safer alternative to other traditional methods, such as ERCP; however, they are diagnostically equivalent [4].

Despite all these pros, MRCP is not flawless. Even in fasting individuals, high‐signal fluids from different abdomen regions can overlap with the pancreaticobiliary system (PBS) fluid signal, limiting the diagnostic accuracy and quality of the MRCP [5]. Slowly moving fluids, such as bile and pancreatic juice, show a high signal on heavily T2‐weighted images. Using negative oral contrast agents enormously shortens T2 and helps to suppress high‐intensity signals in the gastrointestinal tract (GIT). This idea clarifies where the oral contrast agents can reduce unwanted high signals from the GIT [6, 7].

Despite the many oral contrast agents on the market, finding the right one for MRCP remains challenging. The criteria for an accepted oral contrast agent are that the patient should typically accept it, have a uniform distribution in the lumen of the GIT, not be diluted while passing down the track, and not be hazardous to the patient. Also, it should not cause peristalsis, and the cost must be accessible [8]. Contrast agents for MRI of the abdomen are gadopentetate dimeglumine, manganese chloride, kaolinite, antacid, ferric ammonium citrate, barium sulfate, and ferric particles. Either of these falls short due to bad taste, slow digestion in the GIT, cost, or limited market availability [9].

As MRCP has become the best imaging modality for detecting biliary tract diseases, optimizing the patient preparation protocol has become increasingly important. A crucial factor that increasingly affects diagnostic accuracy is preprocedural patient preparation, such as administering a GIT suppression agent, which may improve image quality before MRCP. Hence, it decreases incorrect diagnosis, and the need for additional imaging lowers the rate of negative ERCP and enhances patient care in the future [10]. To date, limited research exists on the effects of preprocedure consumption of specific substances, such as bananas, dark tea, or fasting (4–6 h), on MRCP image quality in patients with OJ. Thus, this study is aimed at comparing MRCP images after using bananas, dark tea, or fasting, as well as to assess the drawbacks of dark tea and bananas in fasting patients for MRCP examination.

## 2. Patients and Methods

### 2.1. Study Design and Setting

This nonrandomized observational cross‐sectional study was conducted on 86 patients with OJ at the imaging center, Sulaimani Teaching Hospital (*n* = 66), and a private center (*n* = 20) in Sulaymaniyah, Iraq, from November 2023 to April 2024. Patients were allocated to preparation groups based on clinical tolerance and preference.

### 2.2. Inclusion Criteria

Patients with OJ who had elevated total serum bilirubin (TSB) and alkaline phosphatase (ALP) were the inclusion criteria.

### 2.3. Exclusion Criteria

Patients who were contraindicated for MRCP imaging, such as those with cardiac pacemakers, cochlear implants, aneurysmal clips, intraocular metallic foreign bodies, or claustrophobia served as the exclusion criteria.

### 2.4. Study Protocol

Both laboratory workup and abdominal tests confirmed the patients′ conditions, and the patients were then referred for MRCP to confirm the further diagnosis. Then, patients′ sociodemographic data and clinical details were recorded. They were divided into three groups (based on their tolerance and preference). Group 1 (*n* = 30) received 115 g of fresh solid banana, Group 2 (*n* = 25) received 250 mL of plain dark tea (without sugar), whereas Group 3 (*n* = 31) received nothing and were fasted for at least 4–6 h before the MRCP examination (they were very ill and could not take anything). Those given banana/dark tea waited for 10 min for the material to coat the stomach/duodenum and suppress their signal intensity. MRCP examinations were performed using 1.5 Tesla MRI scanners (Siemens and Philips), with a T2 HASTE fat‐saturated coronal thick‐slab sequence at both Centers (imaging center at Sulaimani Teaching Hospital and a private center). In the imaging center, a slice thickness of approximately 10 mm, a repetition time of 15, and an echo time of 5 were used. While in the private center, a slice thickness of 3 mm, a repetition time of 155700, and an echo time of 86 were used. The image quality was assessed by the main author (consultant radiologist), who was blinded to the patient preparation groups and clinical data. The reader focused on the anatomy of the intrahepatic duct (IHD), right/left HD, common hepatic duct (CHD), cystic duct, common biliary duct (CBD), and main pancreatic duct (MPD). Additionally, the quality of the GIT suppression signals, pancreaticobiliary tree (PBT) obscuration by other high‐intensity structures (visualization), and the dilatation of different PBT structures were recorded. Image quality was classified as 1–3 for the GIT signals: 1 indicated a signal that partially impedes the analysis of the image findings, 2 indicated a signal that did not hinder the analysis of the image findings, and 3 indicated no signal. Additionally, PBT quality of appearance was scored as 1 (poor) when PBT was mainly obscured, 2 (good) when PBT was partially obscured, and 3 (excellent) when PBT was not obscured [11, 12].

### 2.5. Data Analysis

The IBM Statistical Package for Social Science (SPSS) (Chicago, United States, Version 27) was used for statistical analysis. Descriptive and analytic statistics were performed using the chi‐square test and logistic regression analysis. Odds ratios (ORs) with 95% confidence intervals (CIs) were calculated. A *p* value ≤ 0.05 was considered statistically significant.

## 3. Results

The patients′ ages ranged from 8 to 96 years with a mean of 54.2 ± 21.8 for banana group, 61.6 ± 19 for tea group, and 51.2 ± 20.2 for the group that received nothing (*p* = 0.129). Most patients were aged > 65 years (37.2%) and were female (55.8%). There were no significant differences in age distribution or gender among the preparation groups (*p* > 0.05) (Table 1). The leading cause of obstruction among patients was stones (60.6%), whereas other causes included cholangiocarcinoma, pancreatic head cancer, and postoperative stricture (39.4%).

**Table 1:** Distribution of patients by sociodemographic status.

Variable	Category	Banana	Tea	Nothing	*p* value ^∗^
	Frequency (percentages)				
**Age group (years)**	< 18	2 (6.7)	1 (4.0)	0 (0.0)	0.397
	18–44	8 (26.7)	3 (12.0)	11 (35.5)	
	45–64	9 (30.0)	10 (40.0)	11 (35.5)	
	≥ 65	11 (36.7)	11 (44.0)	9 (29.0)	
**Gender**	Male	13 (43.3)	13 (52.0)	12 (38.7)	0.605
	Female	17 (56.7)	12 (48.0)	19 (61.3)	

^∗^Chi‐square test.

**Figure 1:**
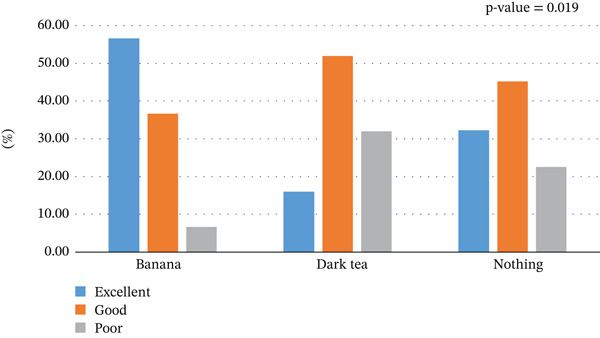
The quality of the pancreaticobiliary tree regarding the preparation used before the examination.

A significant association was observed between preparation type and obscuration of the PBT by high‐intensity structures (*p* = 0.019). Patients who consumed bananas had the highest proportion of “excellent” visualization (56.7%), followed by “good” (36.7%) and “poor” (6.7%). In the plain dark tea group, the majority showed “good” (52%) and “poor” (32%) visualization, whereas the fasting group had lower “excellent” scores (32.3%) and higher “poor” scores (22.6%) (Figure 1).

The visibility of the IHD and extrahepatic biliary structures (right and left hepatic ducts, CHD, cystic duct, and common bile duct) did not differ significantly among the groups (*p* > 0.05). However, a significant difference was observed in the visualization of the MPD, with higher visibility in the banana group (93.3%) compared with plain dark tea (84%) and fasting (67.7%) (*p* = 0.034) (Table 2).

**Table 2:** Visibility of the anatomical structures of the pancreaticobiliary tree in each preparation.

Anatomical structure	Visibility	Banana	Plain dark tea	Nothing	*p* value
	Frequency (%)				
**Intrahepatic duct**	Yes	25 (83.3)	21 (84)	28 (90.3)	0.69
	No	5 (16.7)	4 (16)	3 (9.7)	
**Right and left hepatic duct**	Yes	29 (96.7)	24 (96)	30 (96.5)	0.986
	No	1 (3.3)	1 (4)	1 (3.5)	
**Cystic duct**	Yes	27 (90)	20 (80)	23 (74.2)	0.278
	No	3 (10)	5 (20)	8 (25.8)	
**Common hepatic duct**	Yes	29 (96.7)	25 (100)	30 (96.8)	0.668
	No	1 (3.3)	0 (0.0)	1 (3.2)	
**Common bile duct**	Yes	29 (96.7)	24 (96)	31 (100)	0.554
	No	1 (3.3)	1 (4)	0 (0.0)	
**Main pancreatic duct**	Yes	28 (93.3)	21 (84)	21 (67.7)	0.034 ^∗^
	No	2 (6.7)	4 (16)	10 (32.3)	

^∗^Significant difference using chi‐square test.

Unadjusted ORs were calculated to quantify further association between preparation type and MPD visualization (Table 3). Banana consumption was significantly associated with higher odds of MPD visualization compared with fasting (OR = 7.0, 95% CI: 1.38–35.48, *p* = 0.021). No significant association was observed between plain dark tea and fasting (OR = 2.0, 95% CI: 0.58–6.91, *p* = 0.366). Although banana showed higher odds compared with plain dark tea, this difference did not reach significance (OR = 3.50, 95% CI: 0.62–19.89, *p* = 0.226).

**Table 3:** Crude odds ratios for main pancreatic duct visualization by preparation.

Comparison	Odds ratio (OR)	95% CI	*p* value
**Banana versus fasting**	7.00	1.38–35.48	0.021 ^∗^
**Tea versus fasting**	2.00	0.58–6.91	0.366
**Banana versus tea**	3.50	0.62–19.89	0.226

^∗^Significant difference using chi‐square test.

**Figure 2:**
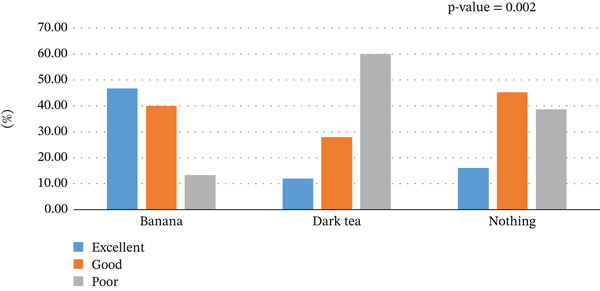
The gastrointestinal tract signal suppression regarding the preparation used before the examination.

**Figure 3:**
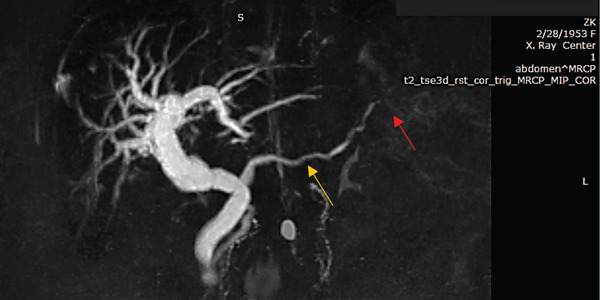
3D T2 HASTE thick slab MRCP‐MIP taken after 10 min of receiving 115‐g banana by the patient. The red arrow shows the excellent depressed signal of the stomach, and the yellow arrow points to the MPD.

**Figure 4:**
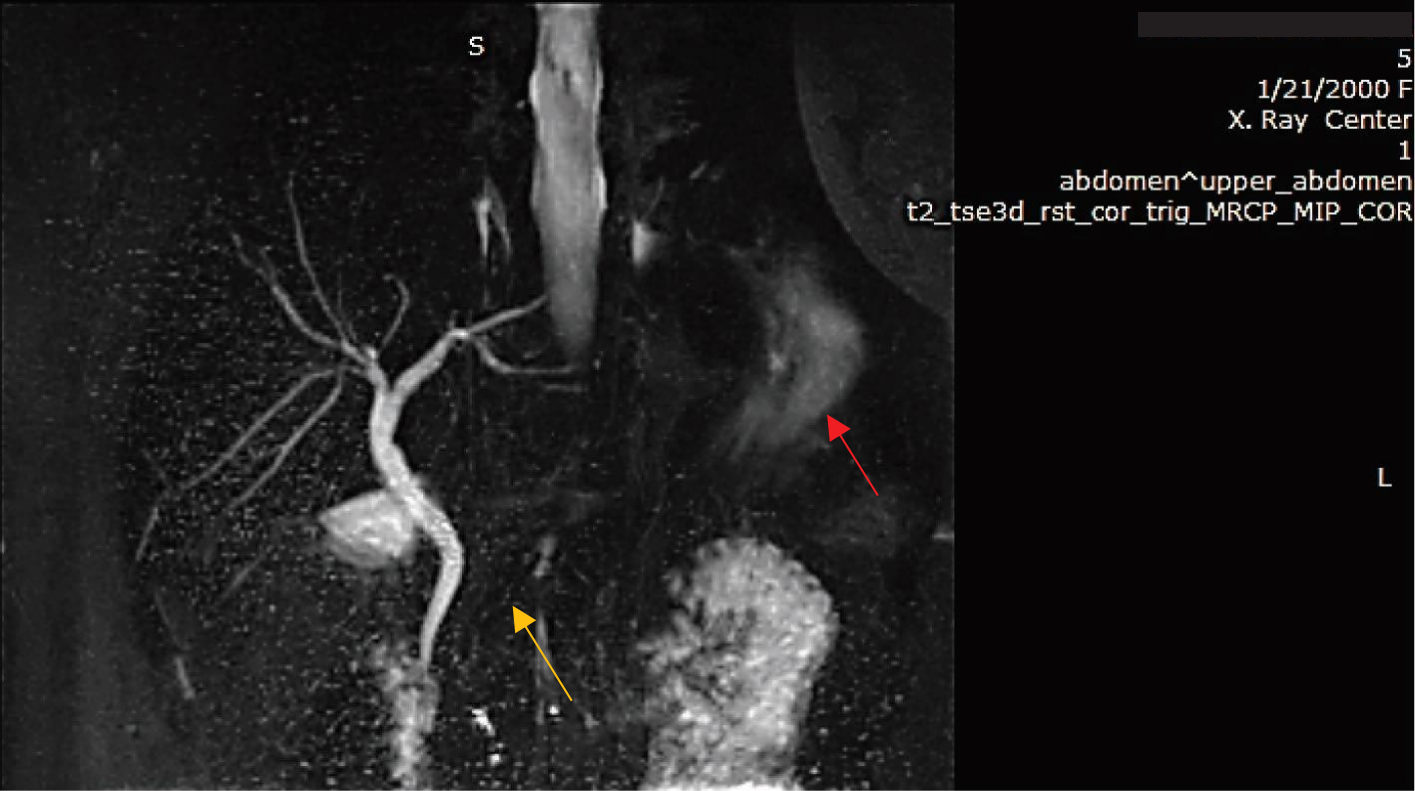
3D T2 HASTE thick slab MRCP‐MIP taken after the patient took nothing. The red arrow shows the good signal suppression of the stomach, and the white arrow points to the MPD.

**Figure 5:**
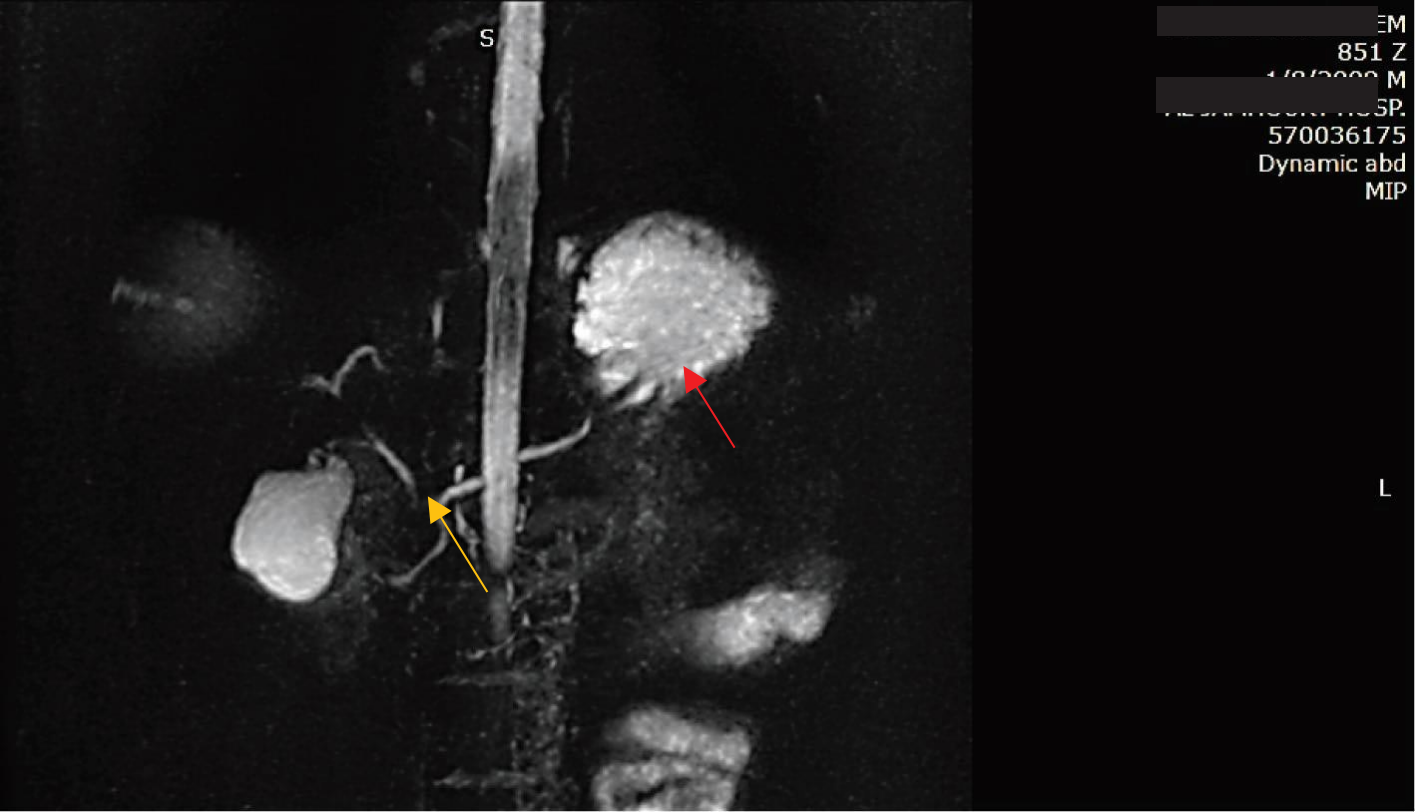
3D T2 HASTE thick slab MRCP‐MIP taken after 10 min of receiving 250‐mL plain dark tea by the patient. The red arrow shows a poorly depressed signal of the stomach, and the yellow arrow points to the MPD.

Significant GIT signal suppression (*p* = 0.002) was observed regarding the preparations used before the MRCP examination (Figure 2). Patients who took a banana had the highest “excellent” score (46.7%), followed by “good” (40%), then “poor” (13.3%) (Figure 3). For plain dark tea, the highest score was “poor” (60%), followed by “good” (28%) and “excellent” (12%) (Figure 4). Patients who took nothing had the highest score for “good” signal suppression (45.2%), followed by “poor” (38.7%), then “excellent” (16.1%) (Figure 5). After all preparations were used, the IHD and extrahepatic biliary tree were mainly dilated (64% and 67.4%, respectively), whereas the MPD was not dilated in most cases (84.9%) (Table 4).

**Table 4:** Assessment of dilation of the pancreaticobiliary tree in different preparations.

Structure	Banana (*n* = 30)	Plain dark tea (*n* = 25)	Nothing (*n* = 31)	*p* value
	Frequency (%)			
**IHD dilation (yes)**	20 (66.7)	14 (58.3)	7 (22.6)	0.001 ^∗∗^
**Extrahepatic dilation (yes)**	19 (63.3)	19 (76.0)	20 (64.5)	0.441
**MPD dilation (yes)**	7 (23.3)	3 (12.5)	13 (41.9)	0.044 ^∗^

Abbreviations: IHD, intrahepatic duct; MPD, main pancreatic duct.

^∗^Significant difference.

^∗∗^Highly significant difference using chi‐square test.

A multivariable logistic regression analysis was performed to adjust for potential confounders, including age and gender (Table 5). After adjustment, banana consumption was significantly associated with improved visualization of the MPD compared with fasting (adjusted OR = 7.00, 95% CI: 1.3–35.0, *p* = 0.019). No significant association was observed for plain dark tea compared with fasting (adjusted OR = 1.87, 95% CI: 0.5–6.9, *p* = 0.338). Age and gender were not significantly associated with MPD visualization.

**Table 5:** Multivariable logistic regression analysis of factors associated with MPD visualization.

Variable	Adjusted OR	95% CI	*p* value
**Banana versus fasting**	7.00	1.3–35.0	0.019 ^∗^
**Tea versus fasting**	1.87	0.5–6.9	0.338
**Age**	0.99	0.96–1.03	0.516
**Gender (female vs. male)**	0.88	0.3–2.5	0.827

^∗^Significant difference using chi‐square test.

No significant associations were found between the appearance quality of the PBS and age or gender (*p* = 0.692 and *p* = 0.868, respectively) (Table 6).

**Table 6:** Assessment of the quality of the pancreaticobiliary tree appearance in different preparations.

Variable		Quality of the pancreaticobiliary tree appearance				
		Poor	Good	Excellent	Total	*p* value
		Frequency (%)				
**Age group (years)**	< 18	1 (33.3)	1 (33.3)	1 (33.3)	3 (100)	0.692
	18–44	5 (23.8)	11 (52.4)	5 (23.8)	21 (100)	
	45–64	4 (13.3)	15 (50)	11 (36.7)	30 (100)	
	≥ 65	7 (21.9)	11 (34.4)	14 (43.8)	32 (100)	
**Gender**	Male	7 (18.4)	18 (47.4)	13 (34.2)	38 (100)	0.868
	Female	10 (20.8)	20 (41.7)	18 (37.5)	48 (100)	

## 4. Discussion

MRCP is a noninvasive imaging technique with many limitations, such as breathing artifacts, overlap of static fluid within the PBS with GIT fluid, and poor spatial resolution. New imaging sequences with thinner sections, shorter acquisition time, and breathing control techniques reduce these limitations [13]. Scientists continuously search for efficient, safe, low‐cost, and readily available negative oral contrast agents for patients before MRCP examinations to improve patient compliance and acceptance of natural contrast, thereby enhancing image quality [14]. Consequently, this study is aimed at determining the optimal MRCP image quality using different periprocedural preparations before the MRCP examination.

MRCP is based on a heavily T2‐weighted sequence to enhance the signal from the fluid of the PBS. The fluid within the GIT has a high signal intensity that may degrade MRCP image quality; thus, patients are advised to fast for 4–6 h before imaging. However, this is still insufficient for eliminating GIT signals [15]. For sufficient elimination of GIT signals, many oral negative contrast agents are available that are rich in high molecular metal ions (iron or manganese), which have paramagnetic or superparamagnetic properties that shorten T2 relaxation time due to the rapid T2 decay and GIT signal darkening. Still, they are either expensive, not palatable, not readily available, or not safe [16]. Nowadays, we have negative oral contrast agents like pineapple, whose natural, pure form is unavailable in many countries, and whose commercial form has not improved MRCP image quality [17]. Pineapple juice is an excellent alternative, but patients should consume 400–500 mL, which is too much for ill patients [11, 12, 18].

Patients were given either banana or plain dark tea (without sugar), or were advised to fast for 4–6 h before the examination to reduce high GIT signals, peristalsis, and motion artifact. Our results showed significant suppression of the GIT signal (*p* = 0.002) and improvement in the appearance quality of the PBS after banana consumption (*p* = 0.019). These results were compatible with those of Utami [13], where banana was used as a preprocedural preparation, and a significant MPD visualization (*p* = 0.02) with dilatation of the extrahepatic biliary tree was observed, thereby making visualization easier.

In this study, visualization of right/left IHD was nonsignificant because they are distant from the stomach and the duodenum, which are not affected by signal intensity, and because the dilation of these ducts makes their visualization easier. The MPD is thin, homogeneous, and obscured by the high signal from the stomach and duodenum due to their proximity, so if not well prepared preprocedurally, it would be challenging to visualize. After banana consumption, there was a significant improvement in MPD visualization (93.3%, *p* = 0.034). However, no significant difference in MPD dilatation was observed. Based on the findings of Ghanaati et al. [19], significant improvements in the visualization of distal CBD (*p* = 0.001) and MPD (*p* < 0.001) were seen that are compatible with this study, in which visualization of the MPD was improved (84%) when plain dark tea was taken as a preprocedural preparation rather than nothing (67.7%). The study is also, consistent with that of Riordan et al. [20], which reported significant GI signal suppression (*p* < 0.001) and improved visualization of the CBD and MPD (*p* < 0.05) after pineapple juice administration. Also, Liang et al. [21] demonstrated that administration of black tea before MRCP significantly reduced GI signal intensity (*p* < 0.001) and improved visualization of the CBD (*p* < 0.01) and MPD (*p* < 0.05), which is compatible with this study.

Multivariable analysis has been performed to adjust potential confounders, and it was found that both age and gender are not significantly related to the appearance quality of the PBS; however, the residual confounding (disease severity and clinical status) might also be possible.

The limitations of this study were the small and unequal sample sizes, the fact that not all patients consumed a fixed volume of plain dark tea because of their intolerance to its bitterness, and the unavailability of other oral contrast agents. Additionally, there was a lack of structured image quality scoring that supports scientific validity. Although multivariable analysis was performed, residual confounding cannot be completely excluded. Also, the nonrandomized design of the study and the use of two scanners with differing protocols represent limitations, as it may introduce potential bias, and the images were assessed by a single reader, so interobserver reproducibility could not be evaluated, and there was a lack of a standardized MRCP imaging protocol because the examinations were conducted in two different centers with different scanner settings and acquisition parameters.

## 5. Conclusions

Banana was found to be an effective oral negative contrast agent due to its high rate of GIT signal suppression, visualization, and PBT dilatation. However, plain dark tea was better for visualizing the MPD than patient‐only fasting. Age and gender were not related to the appearance quality of the PBS. Accordingly, consuming plain dark tea when a banana is unavailable or when the patient has an allergy to it is highly recommended, particularly when MRCP is performed for visualization and assessment of the MPD. Further research with a larger population size and additional preprocedural preparations to obtain the best possible MRCP image quality is suggested.

## Author Contributions

Abeer Kadum Abass Al‐Zuhairy: conceptualization, supervision, and revising the final draft of the manuscript. Sima Radha Hamakhurshid: contributed to data collection, statistical approach, did the data analysis, and writing the manuscript. Sana Radha Hamakhurshid: contributed to study design, data collection, and cultural adaptations, review and assisted in wrting the manuscript. Belan Kamal Muhammad: contributed to the data collection and reviewed and assisted in writing the manuscript, the three authors accept these changes.

## Funding

No funding was received for this manuscript.

## Disclosure

All authors have read and approved the final version of the manuscript.

## Ethics Statement

Approval for the study procedures was granted by the Ethical Committee of the College of Medicine, University of Sulaimani, Sulaimaniyah, Iraq (No. 68 on January 16, 2024). The work was conducted in accordance with the Declaration of Helsinki (2008) and its subsequent amendments.

## Consent

Participants provided written informed consent before starting the study, while their anonymity and confidentiality were protected throughout the study.

## Conflicts of Interest

The authors declare no conflicts of interest.

## Data Availability

The raw and analyzed data from the corresponding author can be provided upon request.
